# Nitrilase GiNIT from *Gibberella intermedia* Efficiently Degrades Nitriles Derived from Rapeseed Meal Glucosinolate

**DOI:** 10.3390/ijms252211986

**Published:** 2024-11-07

**Authors:** Han-Zhi Li, Ming-Yu Liu, Yu-Yue Wang, Xue-Mei Luo, Jia-Xun Feng, Shuai Zhao

**Affiliations:** State Key Laboratory for Conservation and Utilization of Subtropical Agro-Bioresources, Guangxi Research Center for Microbial and Enzyme Engineering Technology, College of Life Science and Technology, Guangxi University, Nanning 530004, Chinaluoxuemei1003@163.com (X.-M.L.)

**Keywords:** nitrilase, nitriles, glucosinolate, rapeseed meal, substrate binding pocket

## Abstract

Rapeseed meal is severely restricted in its utilization as unconventional animal feed due to anti-nutritive compounds, such as glucosinolate, that are degraded to toxic nitriles such as 3-butenenitrile and 4-pentenenitrile in animals. Few studies on nitrilases that can degrade glucosinolate-derived nitriles have been reported thus far. In the present study, a nitrilase gene *GiNIT* from *Gibberella intermedia* was over-expressed in *Escherichia coli* and the purified recombinant nitrilase rGiNIT showed specific activities of 134.48 U/mg and 122.16 U/mg when using 3-butenenitrile and 4-pentenenitrile as substrates at the optimal pH, 7.5, and temperature, 45 °C, which is the highest reported in the literature. The conversion of 3-butenenitrile and 4-pentenenitrile by rGiNIT reached 81.89% and 80.23% after hydrolysis for 15 min and 300 min, respectively. Site-directed mutagenesis and molecular docking analysis revealed that the catalytic ability of rGiNIT depended on the substrate binding pocket comprising 13 key amino acid residues. These results provide a potential enzyme resource for rapeseed meal detoxification and theoretical guidance for protein engineering.

## 1. Introduction

In recent years, the shortage of high-quality feed resources has become increasingly prominent, seriously affecting the development of animal husbandry [1]. Rapeseed meal is the main by-product of rapeseed; with its low price and containing crude proteins accounting for 30–40%, it can be used as a protein feed resource for animal husbandry [2]. Notably, glucosinolate is a kind of anti-nutritional substance present in rapeseed meal that has a negative impact on animal growth and health [3]. Glucosinolate generates toxic nitrile compounds such as 3-butenenitrile and 4-pentenenitrile in the stomach of animals. It is known that 3-butenenitrile and 4-pentenenitrile are harmful to the growth and development of animals [4].

Microbial detoxification is commonly used to remove some toxic compounds, but many fermentation experiments have not quantified the truly harmful compounds resulting from the glucosinolate degradation of rapeseed meal [5]. The existence of 3-butenenitrile and 4-pentenenitrile still seriously limits the application of rapeseed meal as a high-quality feed resource. Traditional physical and chemical reactions use high temperatures and a large number of chemical reagents to degrade nitriles, but may not be suitable for feed applications because of the loss of nutrients, while nitrilase has the advantages of mild reaction conditions and environmental friendliness [6].

Nitrilase (EC 3.5.5.1) is one of the 13 branches belonging to the nitrilase superfamily, and the catalytic triad is commonly composed of Cys-Glu-Lys, which converts nitrile into non-toxic carboxylic acid and ammonia through a hydrolysis reaction [4,5]. Currently, many nitrilases have been explored as green biocatalysts [5,7]. For example, the large-scale production of (R)-mandelic acid is achieved via catalysis with nitrilase [8]. Nitrilase ES-NIT-102 possesses a strong ability to hydroxylate 2-chloroisonicotinic acid to 2-chloroisonicotinic acid [9]. Tang et al. [10] found that 2-chloronicotinonitrile was degraded to 2-chloronicotinic acid by PgNIT from *Paraburkholderia graminis*. Jin et al. [11] recently reported that nitrilase AfNIT from *Acidovorax facilis* could efficiently convert terephthalonitrile into 4-cyanobenzoic acid.

However, the application of nitrilase in agriculture and feed is rarely explored and discussed, especially in rapeseed meal. Only one reference report mentions that the nitrilase BnNIT2 from *Brassica napus* was able to degrade 3-butenenitrile and 4-pentenenitrile, with a specific activity of 58.6 U/mg using 4-pentenenitrile as a substrate [12]. Therefore, it is necessary to identify more nitrilases with the ability to efficiently remove glucosinolate-derived nitriles in rapeseed meal.

In the present study, a nitrilase gene *GiNIT* from the fungus *Gibberella intermedia* was over-expressed in *E. coli* and the purified recombinant nitrilase rGiNIT was found to efficiently degrade 3-butenenitrile and 4-pentenenitrile. Moreover, site-directed mutagenesis and molecular docking analysis were employed to identify the key amino acids for nitrilase catalysis. The enzymatic features of rGiNIT and its mutants were investigated.

## 2. Results

### 2.1. Discovery of Nitrilase with High Hydrolytic Activity Against 3-Butenenitrile and 4-Pentenenitrile

**Figure 1:**
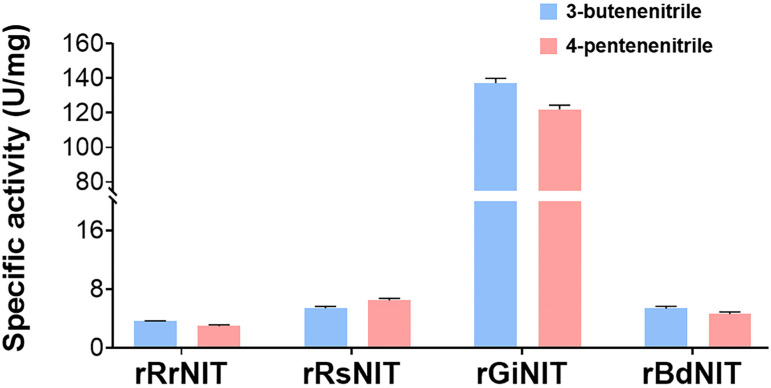
Specific activities of the recombinant nitrilases. The activity is determined with 3-butenenitrile and 4-pentenenitrile as substrates, respectively, at pH 7.5 and 45 °C.

Twelve putative nitrilases annotated in the GenBank database online (Table S1) were selected for study. Artificially synthesized nitrilase genes were cloned and then heterologously expressed in *E. coli* BL21 (DE3). The recombinant nitrilases were purified (Figure S1) and tested for their specific activities against 3-butenenitrile and 4-pentenenitrile, respectively. The results revealed that four purified recombinant nitrilases including rRrNIT from *Rhodococcus rhodochrous* [13], rRsNIT from *Rhodobacter sphaeroides* [14], rGiNIT from *G. intermedia* [15,16] and rBdNIT from *Bradyrhizobium diazoefficiens* [17], exhibited specific activities ranging from 2.98 to 137.36 U/mg. Among them, the recombinant GiNIT (rGiNIT) from *G. intermedia* showed the highest specific activities towards 3-butenenitrile at 137.4 U/mg and 4-pentenenitrile at 122.0 U/mg (Figure 1).

**Figure 2:**
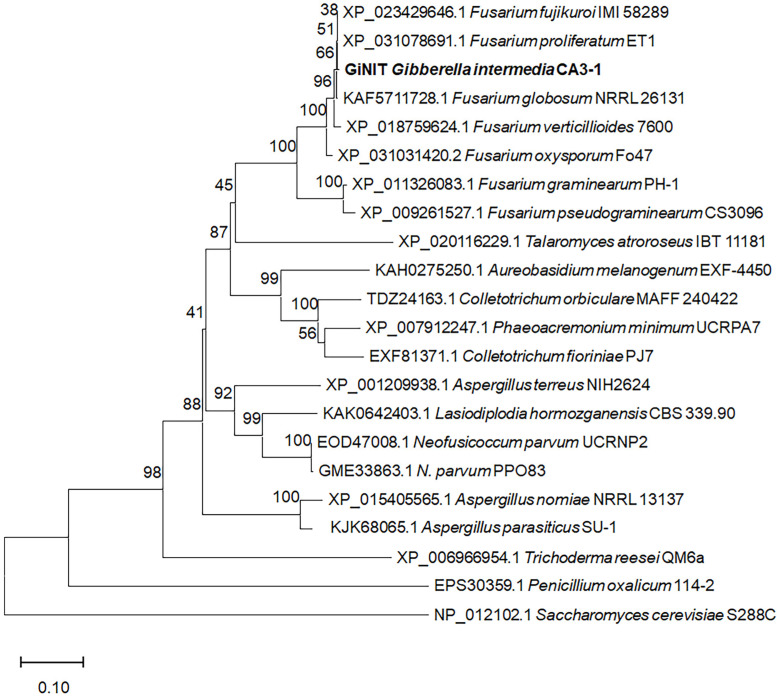
Evolutionary tree of GiNIT and its putative homologs. The cladogram is constructed according to the neighbor-joining method and Poisson model. Bootstrap values are obtained via calculation after setting 1000 replicates and are shown at the nodes.

The GiNIT from *G. intermedia* was reported to be comprised of 320 amino acid residues [16], and shared a high identity of 99.7% with the putative nitrilase (XP_031078691.1) from *Fusarium proliferatum* ET1, but lower than 90% identity with fungal nitrilases from other genera, for example, 66.4% and 52.4% identities with those from *Aspergillus terreus NIH2624* (XP_001209938.1) and *Trichoderma reesei* QM6a (XP_006966954.1), respectively. Evolutionary analysis revealed that GiNIT was very close to those from *Fusarium* (Figure 2). The GiNIT showed high specific activity towards heterocyclic nitriles such as 3- and 4-cyanopyridine [16,18]. However, the degradation ability against nitriles derived from glucosinolate remains unclear. Impressively, this study confirmed that GiNIT possessed the highest specific activity against 3-butenenitrile and 4-pentenenitrile, respectively, in the literature.

### 2.2. Biochemical Characterization and Kinetic Parameters of Nitrilase rGiNIT

**Figure 3:**
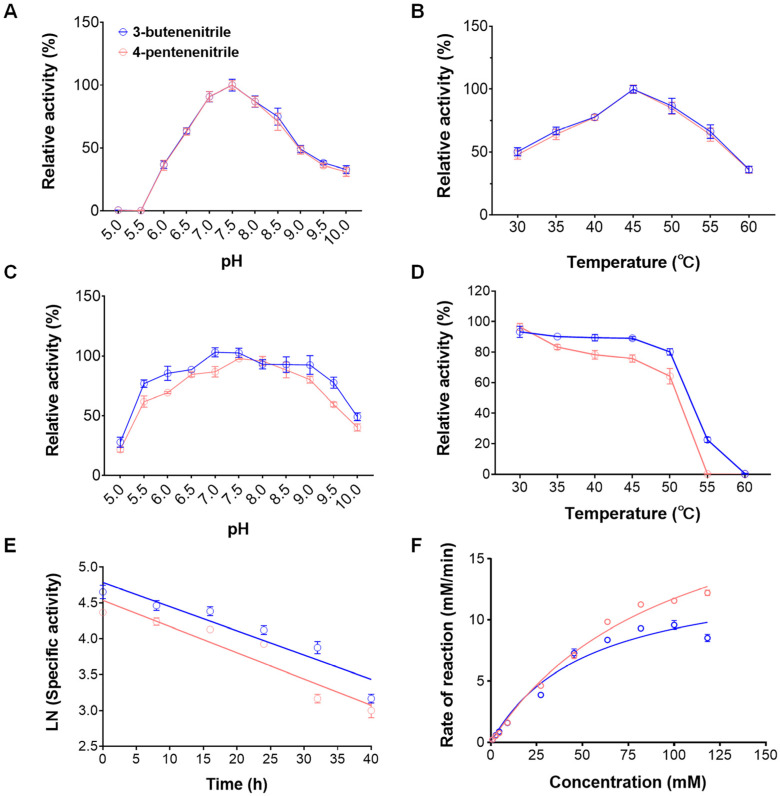
Enzymatic properties of rGiNIT with 3-butenenitrile and 4-pentenenitrile as substrates. (**A**) pH profile of nitrilase activity; (**B**) temperature profile of nitrilase activity; (**C**) effects of pH on nitrilase stability; (**D**) effects of temperature on nitrilase thermostability; (**E**) determination of the half-life of rGiNIT at the optimal pH and temperature; (**F**) determination of Michaelis–Menten constants of rGiNIT. In panels (**A**,**B**), the highest enzyme activity was set as 100%. In panels (**C**,**D**), the specific activity measured for the untreated nitrilase was set as 100%.

The biochemical features of the purified nitrilase rGiNIT were measured. The optimum pH and temperature for rGiNIT activity were 7.5 and 45 °C when using 3-butenenitrile and 4-pentenenitrile, respectively (Figure 3A, B). Interestingly, rGiNIT exhibited excellent stability across a pH range of 6.0 to 9.0 after incubation at 4 °C for 12 h towards 3-butenenitrile. By contrast, when using 4-pentenenitrile as a substrate, rGiNIT maintained over 80% of the original enzyme activity across a pH range of 6.5 to 9.0 (Figure 3C). rGiNIT exhibited relatively good thermostability below 45 °C (Figure 3D). Comparative analysis displayed that rGiNIT showed more pH- and thermo-stability towards 3-butenenitrile than towards 4-pentenenitrile (Figure 3C, D).

Moreover, the deactivation rates and half-life *t*_1/2_ of rGiNIT against 3-butenenitrile and 4-pentenenitrile at 45 °C were determined, respectively. It was calculated that the deactivation rates of rGiNIT were 0.0213 h^−1^ and 0.0243 h^−1^, respectively, while the *t*_1/2_ were 29.52 h and 22.09 h, corresponding to 3-butenenitrile and 4-pentenenitrile (Figure 3E).

The kinetic parameters *Km* and *Vmax* of rGiNIT were 100.3 mM and 23.53 mM·min^−1^ for 3-butenenitrile, and 52.7 mM and 14.16 mM·min^−1^ for 4-pentenenitrile, respectively (Figure 3F).

The influences of metal ions and organic reagents on the nitrilase activity of rGiNIT were investigated. As shown in Table 1, all tested metal ions, except Mg^2+^ and K^+^, exhibited inhibition to different extents. For example, Ag^+^ and Cu^2+^ strongly repressed the nitrilase activity of rGiNIT, resulting in a loss of initial activity of >90%, whereas Ca^2+^, Mn^2+^, and Co^2+^ caused 8.87–20.84% reduced activity. Similarly, all tested organic reagents, especially normal butanol, strongly inhibited the activity of rGiNIT (Table 2).

### 2.3. Efficient Degradation of 3-Butenenitrile and 4-Pentenenitrile by the rGiNIT

**Figure 4:**
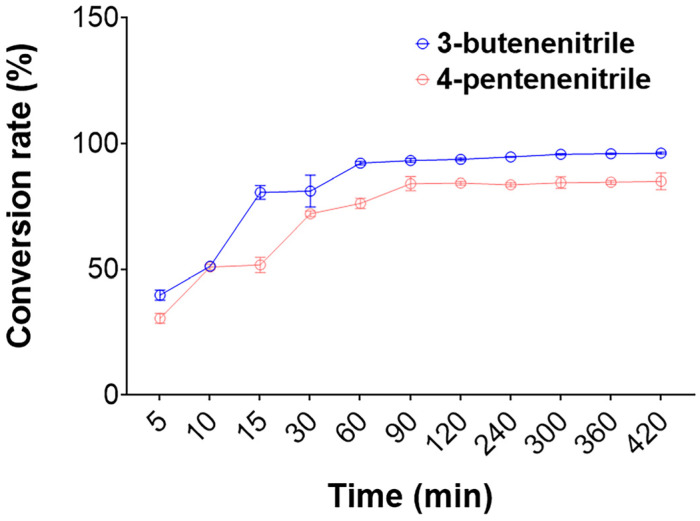
Hydrolysis of 3-butenenitrile and 4-pentenenitrile by the nitrilase rGiNIT. The reaction was performed at pH 7.5 and 45 °C.

The products of nonenzymatic transformations of glucosinolate in rapeseed meal mainly include 3-butenenitrile and 4-pentenenitrile [12]. Therefore, it is necessary to evaluate the degradation ability of rGiNIT towards 3-butenenitrile and 4-pentenenitrile. The results displayed a high conversion of 3-butenenitrile and 4-pentenenitrile into NH_3_ and acids by GiNIT at pH 7.5 and 45 °C. For instance, the conversion rate of 3-butenenitrile reached 92.2% after 1 h of hydrolysis, whereas that of 4-pentenenitrile reached 84.1% after 1.5 h of hydrolysis (Figure 4).

### 2.4. Molecular Docking Analysis of the rGiNIT with Substrates and Determination of Substrate Binding Pocket

**Figure 5:**
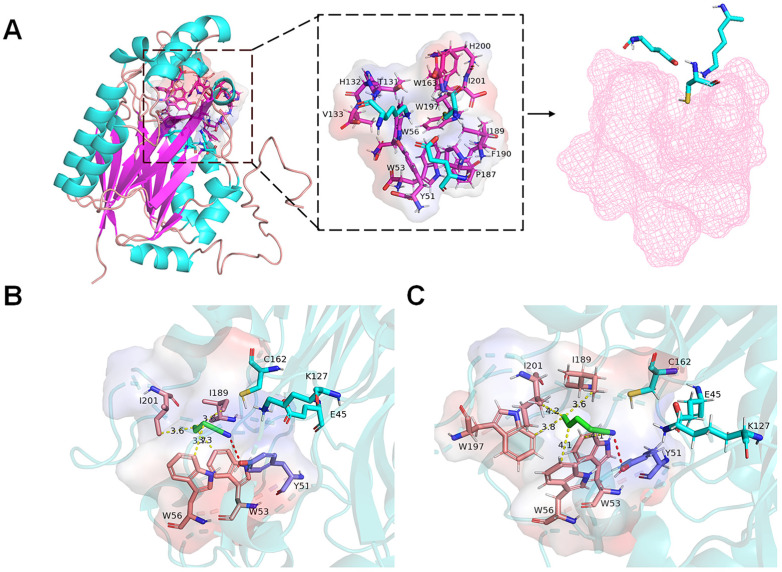
Structure analysis of nitrilase GiNIT (**A**) and molecular docking with 3-butenenitrile (**B**) and 4-pentenenitrile (**C**). In panel (**A**), the 3D structure model of nitrilase GiNIT is predicted with AlphaFold 2.0. Blue amino acid residues constitute the catalytic triad; red amino acid residues constitute the substrate binding pocket; the pink contour is the surface display of the substrate binding pocket. In panels (**B**,**C**), the blue color indicates the substrate molecule.

A previous study indicated that the catalytic triad of GiNIT comprised of E45, K127, and C162 [16]. However, it has been unclear how GiNIT reacts with nitrile via the substrate binding pocket. Software AlphaFold 2.0 was employed to predict the three-dimensional (3D) structure of GiNIT. The results revealed that GiNIT presented a classical sandwich-like α-β-β-α structure. The substrate binding pocket appeared to localize in the loop area between two β-folds, comprising 13 amino acid residues surrounded in 5 Å of the catalytic triad (Figure 5A). Most of the 13 amino acid residues (Y51, W53, W56, T131, H132, V133, W163, P187, I189, F190, W197, H200, and I201) were hydrophobic.

Furthermore, the auto-docking of GiNIT interacting with 3-butenenitrile and 4-pentenenitrile was carried out. The results revealed that Y51 could form hydrogen bonds with 3-butenenitrile and 4-pentenenitrile, respectively. Four amino acid residues, W53, W56, I189, and I201, formed hydrophobic interactions with 3-butenenitrile (Figure 5B), as well as W53, W56, I189, W197, and I201 for 4-pentenenitrile (Figure 5C). As expected, these interacting amino acid residues were localized in the substrate binding pocket.

**Figure 6:**
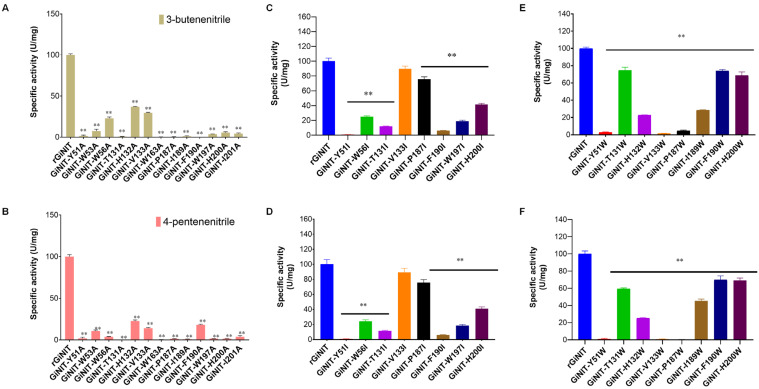
Specific activities of the nitrilase rGiNIT and its mutants. The activity was determined using 3-butenenitrile (**A**,**C**,**E**) and 4-pentenenitrile (**B**,**D**,**F**) as substrates, respectively, at pH 7.5 and 45 °C. ** *p* < 0.01 indicates significant differences between mutants and the wild-type enzyme.

To investigate the effects of amino acid residues in the substrate binding pocket on the nitrilase activity of rGiNIT, Ala-Ile-Trp scanning (AIW-scanning) [19] was performed. When the 13 amino acids were exchanged as alanine, the generated mutants showed reduced enzymatic activities to varying extents toward 3-butenenitrile and 4-pentenenitrile, respectively (Figure 6A, B). For instance, GiNIT-H132A and GiNIT-V133A remained at 11.9%–34.3% of the original activity when using 3-butenenitrile (Figure 6A) and 4-pentenenitrile (Figure 6B) as substrates. Notably, the specific activity of both of them towards 3-butenenitrile was considerably higher than those on 4-pentenenitrile. The other mutant nitrilases almost lost nitrilase activities (Figure 6). Similarly, when isoleucine (Ile) and tryptophan (Trp) substituted the tested amino acids, respectively, the resulting mutants showed reduced specific activities by 10.5%–99.5% relative to the wild-type enzyme, except GiNIT-V133I, which showed no significant alteration (Figure 6C–F). Therefore, those results suggest that all amino acid residues in the substrate binding pocket are required for nitrilase activity.

**Figure 7:**
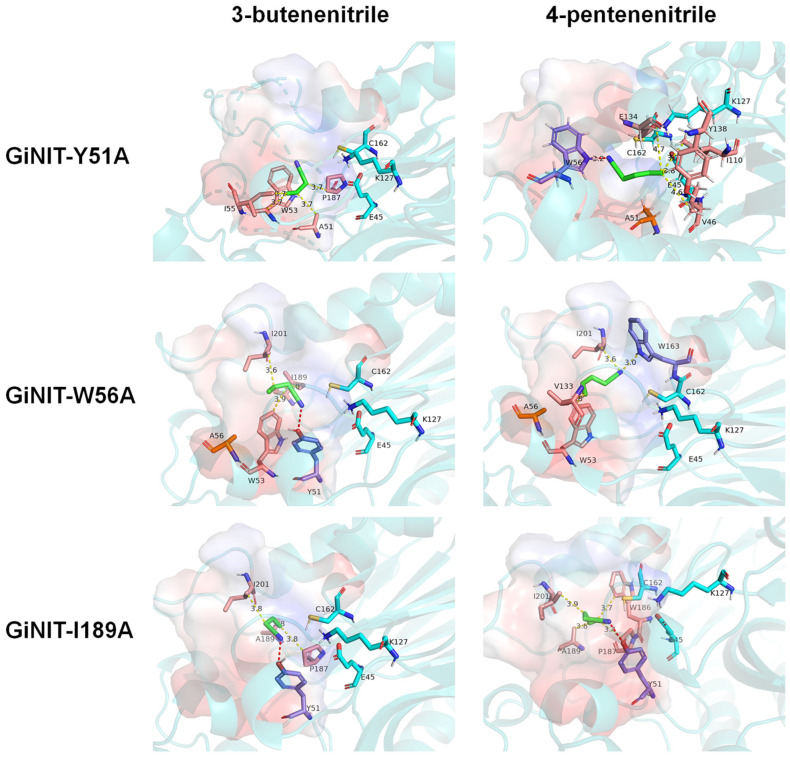
Molecular docking of nitrilase mutants with 3-butenenitrile and 4-pentenenitrile.

After the key amino acid Y51 was mutated to alanine, there was no hydrogen bond formed when the mutated enzyme GiNIT-Y51A docked with 3-butenenitrile, and the position of substrate binding deviated from the native substrate binding pocket to the catalytic triad. When GiNIT-Y51A docked with 4-pentenenitrile, the amino acid forming a hydrogen bond became W56, which is far away from the catalytic triad. The docking results of mutant nitrilases GiNIT-W53A, GiNIT-T131A, GiNIT-W163A, GiNIT-P187A, and GiNIT-F190A were similar to that of GiNIT-Y51A (Figure S2). When GiNIT-W56A docked with 3-butenenitrile, it formed hydrogen bonds with amino acid residue Y51, and the position of binding to the active pocket was close to K127. Differently, when GiNIT-W56A docked with 4-pentenenitrile, no hydrogen bond was formed (Figure 7). The molecular docking of the mutant nitrilases GiNIT-H132A and GiNIT-V133A with substrates displayed similar results to that of GiNIT-W56A (Figure S2). Therefore, it was speculated that the position of the substrate binding to the active pocket crucially affected the nitrilase activity.

Notably, amino acid residue Y51 from GiNIT-I189A could form a hydrogen bond with 3-butenenitrile, and the substrate was also localized in the native substrate binding pocket. However, it was found that the mutation of I189 into alanine caused a conformation alteration in the binding pocket, resulting in unstable binding with the substrate (Figure 7). In addition, compared with rGiNIT, the number of key amino acids in the hydrophobic interaction of mutants GiNIT-W197A, GiNIT-H200A, and GiNIT-I201A with the two substrates decreased, which might lead to the instability of the structure of the substrate binding pocket (Figure S2).

## 3. Discussion

Nitrilases, as potential green biocatalysts, play a pivotal role in catalyzing the degradation of nitriles into nontoxic carboxylic acid and ammonia, which has garnered great attention in multiple industries [4]. Glucosinolate-derived nitrile compounds, as anti-nutritive factors, seriously limit the application of rapeseed meal in animal feed [20]. However, few nitrilases have been explored for rapeseed meal detoxification. In the present study, we found that the nitrilase GiNIT from *G. intermedia* exhibited highly specific activities and efficient degradation ability against 3-butenenitrile and 4-pentenenitrile, which are derived from the nonenzymatic transformation of glucosinolates in rapeseed meal. Moreover, the enzymatic characterization of GiNIT and its active pocket were investigated. These present results provide a potential enzyme resource for rapeseed meal detoxification and novel insights into the protein engineering of nitrilases.

A previous report revealed that the GiNIT (formerly GICA31) from *G. intermedia* showed high specific activity towards heterocyclic nitriles, for instance, with an activity of 0.5 U/mg dry cell when hydrolyzing 3-cyanopyridine [16]. However, the degradation ability against 3-butenenitrile and 4-pentenenitrile remains unclear. Impressively, it was found here that the GiNIT possessed the highest specific activity against 3-butenenitrile and 4-pentenenitrile, reaching 134.48 U/mg and 122.16 U/mg, respectively. In only one report, a plant nitrilase, BnNIT2, exhibited a specific activity of 44.5 U/mg and 58.6 U/mg towards 3-butenenitrile and 4-pentenenitrile, respectively [12].

Accumulated evidence from sequence alignment analysis finds a conserved catalytic triad consisting of Cys-Glu-Lys for the action of nitrilases across bacteria, fungi, animals, and plants. The catalytic triad of GiNIT involves E45, K127, and C162 [16]. To date, the broadly accepted catalytic mechanism of nitrilase includes several steps, such as substrate binding, nucleophilic attack, proton transfer, and product release. Among them, substrate binding is recognized as a pivotal process [4]. However, the binding pocket of nitrilase exhibits substrate-dependent diversity.

This study confirmed that the 13 amino acids around the catalytic triad of GiNIT constituted its substrate binding pocket and are required for its specific activity. These residues appear to be associated with the formation of hydrogen bonds and hydrophobic interactions between enzyme and substrate molecules, substrate localization, or conformation of the binding pocket. In addition, the size of the substrate binding pocket is very important for nitrilase catalytic ability. Commonly, a big pocket facilitates the entry of substrate molecules, but impacts the stability of enzyme–substrate complexes. Nevertheless, a small pocket hinders the binding of the enzyme with the substrate [21]. Comparative analysis indicates that the substrate binding pocket of GiNIT (296.19 Å^3^) is significantly smaller than that of BnNIT2 (1005.46 Å^3^) when binding 3-butenenitrile and 4-pentenenitrile, which is a possible reason for the high specific activity. Moreover, the double mutant V198L-W180G from *Synechocystis* sp. PCC6803 significantly reduces the ability of hydrolyzing 3-chloropropanonitrile due to the expansion of the active pocket leading to the escape of the substrate [22]. In fact, attempts were made to shrink the active pocket of GiNIT, aiming to improve enzymatic activity against 3-butenenitrile and 4-pentenenitrile. However, the activity of the resulting mutants indicates reduction, suggesting that the native active pocket is suitable for the entry of substrate molecules.

In addition, the stability of enzyme–substrate interaction and the distance of the substrate to the catalytic triad are crucial for nitirlase catalytic efficiency. For instance, mutant Y51A lost the formation of a hydrogen bond with 3-butenenitrile; by contrast, when docking with 4-pentenenitrile, the position of substrate binding was far from the catalytic triad. Aguirre-Sampieri et al. [21] propose that during the reaction, the catalytic cysteine is activated by glutamic acid through a bridging water molecule. Then, the nucleophilic attack happens by the activated thiol group on the cyanocarbon atom of the substrate; simultaneously, the proton transfers from the substrate to the lysine, forming a thioimidate intermediary. Subsequently, the tetrahedral intermediate forms due to the attraction by the water molecule on the cyanocarbon atom in the nitrile. Therefore, a strongly stabilized enzyme–substrate complex and a reasonable distance between the substrate molecule and the catalytic triad facilitate the catalytic reaction.

In addition, it should be noted that the hydrolysis of 3-butenenitrile and 4-pentenenitrile by nitrilases, including GiNIT, produces 3-butenoic acid and 4-pentenoic acid, respectively, in addition to ammonia. Of them, ammonia is utilized as a nitrogen source. 3-Butenoic acid apparently increases the fermentation and digestion of feed [23]. The Research Institute for Fragrance Materials (RIFM) safety assessment demonstrates that 4-pentenoic acid exhibits no skin sensitization potential and genotoxicity [24]. However, both 3-butenoic acid and 4-pentenoic acid cause irritation to a certain degree, and whether they are really safe for animals still requires further assessment.

## 4. Materials and Methods

### 4.1. Microorganism Strains and Cultivation

*Escherichia coli* DH5α and BL21 (DE3) were used as the cloning and expression hosts, respectively, and they were cultivated in Luria–Bertani (LB) medium at 37 °C.

### 4.2. Expression and Purification of the Recombinant rGiNIT

Gene *GiNIT*, synthesized artificially, was cloned into the vector pET-32a(+). The constructed recombinant plasmids were transformed into *E*. *coli* BL21 (DE3). The recombinant strain was grown in LB medium containing 50 μg/mL ampicillin (Solarbio Life Science, Beijing, China) at 37 °C for 3–6 h. Isopropyl-β-D-thiogalactoside (0.5 mM, Solarbio Life Science) was added to induce gene expression until the optical density of the culture reached OD_600_ of 0.8. Subsequently, the culture was incubated at 16 °C for 20–24 h. The *E*. *coli* cells were collected by centrifugation at 8000 rpm and 4 °C for 10 min. The Ni-NTA protein purification column (Beyotime Biotechnology, Shanghai, China) was used to purify the recombinant protein. The proteins were detected by sodium dodecyl sulfate-polyacrylamide gel electrophoresis (SDS-PAGE).

### 4.3. Determination of Nitrilase Activity

The specific activity of nitrilase was tested in phosphate buffer saline (pH 7.5) containing 50.0 mM of 3-butenenitrile or 4-pentenenitrile (Shanghai Aladdin Biochemical Technology, Shanghai, China) and a certain amount of recombinant nitrilase. The reaction was stopped by adding 100 μL of 2 M HCl after being placed at 45 °C for 15 min. The amount of ammonia released was measured by the Bertholet assay [25]. One unit (U) of the nitrilase activity was defined as the enzyme amount required to produce 1 μmoL of ammonia per minute.

### 4.4. Biochemical Characterization of Nitrilase

The optimal pH and temperature of nitrilase were determined with pH and temperature ranging between 5.0–10.0 and 30–60 °C, respectively. The maximum activity of the enzyme observed was defined as 100%. The effects of pH and temperature on enzymatic stability were investigated by incubation at pH 5.0–10.0 for 12 h and 30–60 °C for 1 h, respectively. The residual specific activity of the enzyme was determined under the optimum pH and temperature. The hydrolysis rate by rGiNIT toward 50.0 mM of 3-butenenitrile and 4-pentenenitrile was determined within 420 min at the optimal pH and temperature.

### 4.5. Effect of Metal Ions and Organic Reagents

Various metal ions (Ag^+^, Fe^3+^, Mg^2+^, Ca^2+^, Fe^2+^, Zn^2+^, Mn^2+^, Cu^2+^, Co^2+^, K^+^) (Solarbio Life Science) at concentrations of 1 mM and organic reagents (methanol, ethanol, ether, chloroform, isopropanol, and N-butanol) at concentrations of 5% (*v*/*v*) and 20% (*v*/*v*) were evaluated to determine their effects on the enzymatic activity of rGiNIT.

### 4.6. Bioinformatics Analysis of rGiNIT

The protein structure of GiNIT was predicted by AlphaFold 2.0 [26]. The 3D structure of the substrate was obtained from the PubChem database (https://pubchem.ncbi.nlm.nih.gov/; accessed on 17 September 2022). AutoDock Vina [27] was used to dock the substrate molecule to the nitrilase protein. The evolutionary tree was constructed by software MEGA 11 [28] according to the neighbor-joining method and Poisson correction model. Bootstrap values were obtained by calculation after setting 1000 replicates.

### 4.7. Statistical Analysis

Statistical analysis of the experimental data was carried out with Microsoft Excel (Office 2019, Microsoft, Redmond, WA, USA), using a two-tailed Student’s *t* test.

## 5. Conclusions

This study obtained the nitrilase GiNIT with the highest specific activity towards 3-butenenitrile and 4-pentenenitrile as substrates reported in the current literature. For the first time, the amino acids that constitute the substrate binding pocket of nitrilase GiNIT were identified as the key amino acids affecting enzyme activity. GiNIT was shown to rapidly eliminate 3-butenenitrile and 4-pentenenitrile into nontoxic carboxylic acids, which is a green method for the detoxification of glucosinolate in rapeseed meal.

## Figures and Tables

**Table 1:** Effects of metal ions on activity of recombinant nitrilase rGiNIT.

Metal Ions (5 mM)	Relative Activity with 3-Butenenitrile as Substrate (%)	Relative Activity with 4-Pentenenitrile as Substrate (%)
Control	100 ± 0.63	100 ± 0.53
Ag^+^	2.30 ± 0.10	4.20 ± 0.68
Fe^3+^	57.98 ± 0.39	54.18 ± 0.98
Fe^2+^	50.68 ± 1.72	50.98 ± 1.67
Mg^2+^	102.51 ± 1.72	103.14 ± 0.38
Ca^2+^	88.29 ± 1.31	79.16 ± 0.07
Zn^2+^	71.35 ± 1.56	76.73 ± 1.69
Mn^2+^	88.57 ± 2.51	91.13 ± 0.61
Cu^2+^	11.28 ± 0.68	8.31 ± 0.83
Co^2+^	91.65 ± 1.28	89.45 ± 2.77
K^+^	100.80 ± 0.31	96.75 ± 1.27

**Table 2:** Effects of organic reagents on activity of recombinant nitrilase rGiNIT.

Organic Reagents		Relative Activity with 3-Butenenitrile as Substrate (%)	Relative Activity with 4-Pentenenitrile as Substrate (%)
control		100 ± 0.96	100 ± 0.98
methanol	5%	50.05 ± 1.70	57.08 ± 0.87
	20%	3.48 ± 0.06	2.71 ± 0.15
ethanol	5%	40.07 ± 3.35	55.53 ± 2.01
	20%	3.20 ± 0.21	2.59 ± 0.31
ether	5%	61.63 ± 0.79	93.85 ± 1.19
	20%	62.48 ± 1.19	71.85 ± 0.86
chloroform	5%	65.03 ± 3.37	88.01 ± 0.72
	20%	27.59 ± 3.18	27.91 ± 0.35
isopropanol	5%	34.46 ± 1.50	55.53 ± 0.69
	20%	3.58 ± 0.26	9.74 ± 0.12
normal butanol	5%	7.02 ± 0.12	2.59 ± 0.23
	20%	4.14 ± 0.06	2.94 ± 0.32

## Data Availability

The datasets and materials used and/or analyzed during the current study are available from the corresponding author upon reasonable request.

## Supplementary Materials

The supporting information can be downloaded at https://www.mdpi.com/article/10.3390/ijms252211986/s1.
