# Supplementary material for: Nitrilase GiNIT from Gibberella intermedia Efficiently Degrades Nitriles Derived from Rapeseed Meal Glucosinolate
Source: Int J Mol Sci. 2024 Nov 7;25(22):11986. doi: 10.3390/ijms252211986 (PMC11594246; doi:10.3390/ijms252211986)
Supplement: Supplementary file 1 [file ijms-25-11986-s001.zip › ijms-3266819-supplementary.pdf]

International Journal of
Molecular Sciences

MDPI

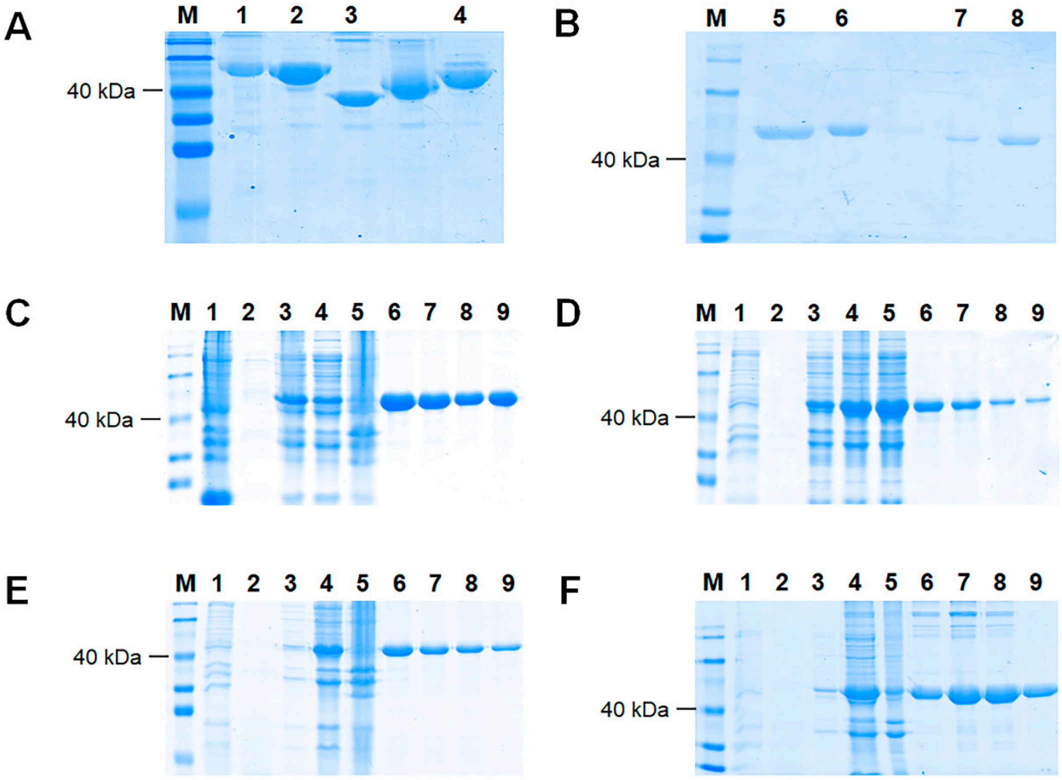

Figure S1. Sodium dodecyl sulfate-polyacrylamide gel electrophoresis (SDS-PAGE) analysis of the recombinant nitrilases. (A-B) Purified nitrilases. M: Marker; 1: rBdNIT; 2: rRrNIT; 3: rGiNIT; 4: rRsNIT; 5: rPpNIT; 6: rPaNIT; 7: rRlNIT; 8: rPfNIT. (C) Purification of rPgNIT1. (D) Purification of rRaNIT. (E) Purification of rAoNIT. (F) Purification of rPtNIT. In panels C-F, M: Marker; 1: Supernatant of bacterial cells carrying blank plasmid after lysis; 2: Supernatant of culture after centrifugation; 3: Liquid solution of recombinant bacterial cells after lysis; 4. Supernatant of recombinant bacterial cells after lysis; 5: Precipitates of recombinant bacterial cells after lysis; 6-9: Purified recombinant nitrilases.

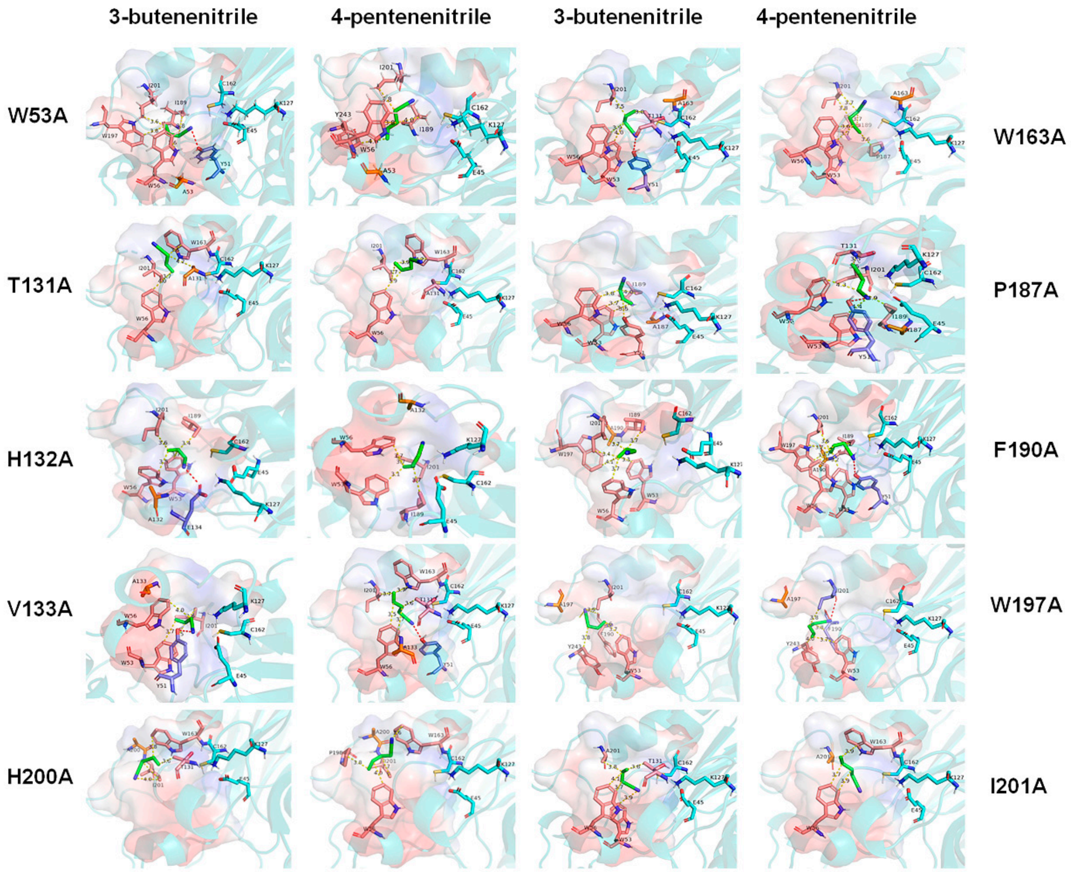

Figure S2. Molecular docking of nitrilase mutants with 3-butenenitrile and 4-pentenenitrile.

Table S1. The nitrilase genes selected for this study.

Name	Source	GenBank accession number/ References	GenBank accession number of protein/ References
PpNIT	*Pseudomonas poae*	NZ_LT629706.1	WP_003232792.1
RlNIT	*Rhizobium laguerreae*	NZ_JAAXQT010000010.1	WP_221988741.1
PfNIT	*Pseudomonas fragi*	NZ_CP064354.1	WP_016781301.1
PaNIT	*Pseudomonas asiatica*	NZ_BLJF01000001.1	WP_015271513.1
PgNIT1	*Parabacteroides goldsteinii*	NZ_CP081906.1	WP_007655830.1
RaNIT	*Rhizobium anhuiense*	NZ_BMFI01000002.1: 75108-75953	WP_097630921.1
AoNIT	*Alistipes onderdonkii*	NZ_AP019734.1	WP_087403418.1
PtNIT	*Parageobacillus thermoglucosidasius*	NZ_CP016622.1	WP_013401820.1
RrNIT	*Rhodococcus rhodochrous*	D12583.1	BAA02127.1 [13]
RsNIT	*Rhodobacter sphaeroides*	JN635494.1	AEP68093.1 [14]
GiNIT	*Gibberella intermedia*	[15]	[16]
BdNIT	*Bradyrhizobium diazoefficiens*	NZ_AP022638.1: 4174468-4175433	WP_011086181.1 [17]
